# Expression of pS2 in prostate cancer correlates with grade and Chromogranin A expression but not with stage

**DOI:** 10.1186/1471-2490-4-14

**Published:** 2004-12-10

**Authors:** M Hammad Ather, Farhat Abbas, Nuzhat Faruqui, M Israr, Shahid Pervez

**Affiliations:** 1 Dept. of Surgery, Aga Khan University, Karachi, Pakistan; 2 Dept. of Pathology, Aga Khan University, Karachi, Pakistan

## Abstract

**Background:**

The biological potential of prostate cancer is extremely variable. Particular interest is focused on markers not expressed in normal prostatic tissues. pS2 protein expression has been demonstrated in a range of malignant tissues in an oestrogen-independent pathway. Recently, it has been demonstrated that pS2, in prostate cancer, is closely associated with neuro-endocrine differentiation. In the present study, we have analyzed, the potential of Neuro-endocrine and pS2 (TFF1) expression in human prostate cancer determined by immunohistochemistry, in primary adenocarcinoma of the prostate and attempted to correlate this with the clinico-pathologic features of the patient and neuroendocrine expression.

**Methods:**

Ninety-five malignant prostatic specimens from primary adenocarcinoma, obtained from either transurethral resection of prostate or radical retropubic prostatectomy, from 84 patients between January 1991 and December 1998 were evaluated by immuno-histochemical staining using selected neuroendocrine tumor markers i.e. chromogranin A (CgA) and estrogen inducible pS2 protein. The relationship between the expressions of pS2 was studied with CgA expression, clinical stage (TNM) and tumour grade (Gleason system). Fischer exact test was used for statistical analysis.

**Results:**

The mean age of the patients was 70 + /- 9.2 years. The pS2 expression was seen in 10% of primary prostate cancers. Worsening histological grade was associated with greater expression of pS2 (p < 0.001). The expression of CgA was noted in 31% of malignant prostatic tissue. In pS2, positive cases 2/3rd of patients were also CgA +ve. However, there was no significant correlation between pS2 expression and the stage of disease.

**Conclusion:**

pS2 expression in prostate cancer significantly correlates with histological grade and the neuroendocrine differentiation, as demonstrated by Chromogranin A expression but not with the clinical stage of the disease. However, the overall expression was low consequently; no definitive conclusions can be drawn. We feel further work is required in a larger series, both in primary and metastatic cancer.

## Background

The biological potential of prostate cancer is extremely variable [1]. It is perhaps the only cancer, which could be managed by, deferred treatment in its early course for selected cancers [2]. To define the biological potential of prostate cancer, prognostic markers are employed. There are numerous markers for assessing the biological aggressiveness of the prostate cancer [3]. However, large studies have shown that they lack sensitivity and specificity due primarily to their expression in normal prostatic epithelium as well. This justifies a recent surge in interest in markers specific to malignant prostatic tissue [4]. Recent studies have shown the potential of neuro-endocrine differentiation in adenocarcinoma of the prostate and its role in ascertaining the biological aggressiveness of the tumor [3]. Wang et al [5] has recently noted that the expression of the pS2 protein is implicated in the pathogenesis and progression of some neuro-endocrine tumors.

Maisakowski et al. first described the pS2 gene in the MCF-7 human breast cancer cell line [6]. The pS2 is a cysteine rich secretory protein, containing 84 amino acids and a molecular weight of 6.45 k-Da. The pS2 gene is highly expressed in estrogen-receptor positive breast cancer, and high levels of pS2 protein correlate with responsiveness to primary endocrine therapy and better patient survival in breast cancer. However, in prostate cancer it is linked with NE differentiation and poorer outcome [7].

In the present study, we have investigated the expression of pS2 in malignant primary prostatic tissue in specimens obtained from transurethral, open prostatectomy, and correlated this with neuro-endocrine differentiation and clinical stage and grade. This is a preliminary report on pS2 expression in prostate cancer, a larger study will better define the correlation between stage, grade of cancer with pS2 and CgA expression.

## Methods

### Demographic profile

Ninety-five malignant consecutive primary prostatic specimens were obtained from 84 patients by either trans-urethral resection of prostate (n = 69 patients) for urinary obstruction or from radical retro-pubic prostatectomy (n = 15 patients) between January 1991 and December 1998. These tissue specimens were taken from the archived records of the department of pathology. The age ranged from 52–93 years (mean 70 + 9.2 years).

Immuno-histochemical staining for pS2 and Chromogranin A Sections were stained for H & E as well as for pS2 (Novocastra, UK Cat. # NCL-pS2) and Chromogranin A (DAKO, Glostrup, Denmark Cat # A0430) by immunohistochemistry using indirect immunoperoxidase technique.

Briefly, 3 μm thick tissue sections were cut and mounted on poly-L-lysine (sigma) coated slides. Sections were deparaffinized in xylene and re-hydrated through graded alcohol series followed by water. Antigen retrieval was done in case of pS2 with 10 mM citrate buffer, 6.0 in a microwave oven 3 × 5 seconds at 450 W, then gradually cooled down to room temperature.

Sections were washed with water followed by Phosphate buffer saline (PBS) rinse.

Endogenous peroxidase in the sections was blocked for 30 minutes with 0.3% H2 02 in methanol. Sections were washed with PBS. All sections were treated with Normal Swine serum (NSS) prediluted 1:10 in PBS for 5 minutes. The sections were then incubated with the primary antibody to pS2 diluted with NSS (1:100) and Chromogranin A (1:20) for 90 minutes at room temperature. Slides were washed with PBS and incubated with peroxidase-conjugated swine anti rabbit secondary antibody (DAKO) at a dilution of 1:150 for 45 minutes at room temperature. 3, 3'-diaminobenzidine (DAB) was used as a final Chromogen. Harris Haemtoxylin was used as a counter nuclear stain. Positive and negative controls were used with all batches of IHC staining. A prostatic adenocarcinoma specimen section expressing pS2 was used as a positive control. Same case exhibiting the primary antibody was used a negative control with each staining procedure. The extent of pS2 reactivity was semi quantitatively assessed by estimating the percentage of positive acini present in the whole mounted sessions. Expression was graded ++ if more than 50% of the tissue showed expression, + if between 5 and 49% showed expression and focal if <5% showed expression.

Histological grading The Gleason system was used for grading of the cancer specimens; a senior histopathologist (SP) blinded of previous Gleason grading and clinical course did this. Based upon the Gleason score patients were divided into three groups i.e. well differentiated (Gleason sum 2–4), moderately differentiated (Gleason sum 5–7) and poorly differentiated (Gleason sum 8–10).

To study correlation and determine the p value Student t test was applied.

## Results

The cancerous lesion composed of 35% (n = 29) stage T1, 32% (n = 27) stage T2, 25% (n = 21) stage T3 and 6% (n = 5) stage T4 disease according to the TNM classification. Based upon the stage of the disease patients were divided into three groups i.e. organ confined (T1-2), locally invasive (T3-4 and N1) and metastatic (M1) cancer.

**Figure 1:**
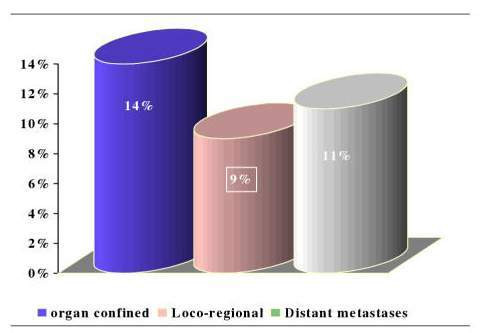
Expression of pS2 % in organ confined, locally advanced (locoregional) and metastatic adenocarcinoma of the prostate.

In 95-cancer specimen from transurethral resection (n = 69) for urinary obstruction and radical retropubic prostatectomy for organ-confined cancers (n = 15), pS2 reactivity was detected in the adjoining normal or hyperplastic acini in only 4.2%. The pS2 expression in cancer was found in 10% (figure 1). The immuno-histochemical reactivity of pS2 in malignant epithelial cells was confined to the cytoplasm of with a tendency to a perinuclear accentuation.

Expression of pS2 was correlated with the stage of disease in Figure 1. Staining for NE marker (CgA) was seen in 31% (figure 1); correlation between the pS2 and CgA expression is summarized in table 1, it showed that 2/3rd of pS2 also showed CgA expression.

**Figure 2:**
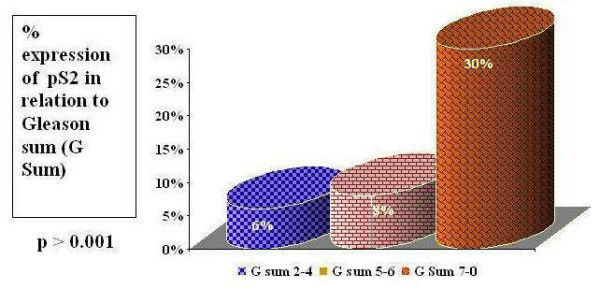
Correlation of Gleason sum and pS2 expression.

**Figure 4:**
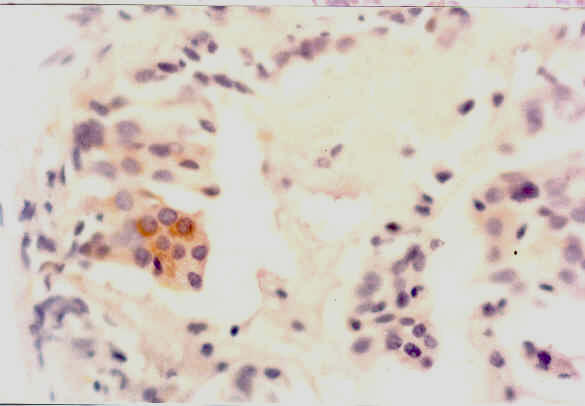
Photomicrograph of prostatic adenocarcinoma stailed with a polyclonal against pS2. Note prominent perinuclear brown staining in tumour cells. Magnification: 40×

Worsening histological grade was associated with greater expression of pS2. In Gleason sum groups 2–4 and 5–6, expression of pS2 was noted in 6 and 8% respectively whereas in Gleason sum group 8–10 the expression was observed in 30% (p < 0.001). The expression of pS2 [figure 4(a) and 4(b)] in various prognostically and therapeutically distinct groups based upon the grade of cancer is described in figure 2.

## Discussion

In the present study, we investigated the expression of pS2 protein in the adenocarcinoma of prostate and in the surrounding normal prostate tissue. We used a standard immunohistochemical method to assess pS2 expression in tissue sections of adenocarcinoma prostate instead of instead of biochemical or immuno-radiometric assay. The immuno-histochemical method for detection of pS2 expression has drawbacks in comparison to biochemical and immunoradiometric assay on tissue extracts. Both of the later methods allow precise quantification of levels of expression for a better correlation with other parameters studied.

However, as we are interested in the clinical utility of pS2 expression in our prostate cancer population, we used immunohistochemistry, which allows appreciation of intra-tumoral heterogeneity of expression and of both cancerous and non-cancerous cells. pS2 protein expression has been demonstrated in a range of malignant and benign pathologies. It is highly expressed in receptor positive human breast cancer [5] but expression in other cancers like ovarian [7], cervical [8], gastrointestinal [9], thyroid [10] and bladder [11] is variable.

A significant implication of pS2 in prostate cancer is the close association of this marker with Neuroendocrine (NE) differentiation. There is increasing evidence that focal NE differentiation frequently occurs in prostatic adenocarcinoma and it may have significant prognostic implications [12-14]. NE differentiation is also described in hormone refractory prostate cancer; Krijnen et al [14] noted that androgen receptors are not present in prostatic adenocarcinoma staining positive for CgA. While Higashiyama noted 17% expression of pS2 in all pulmonary cancers, Wang et al [5] noted 45% expression in small cell cancers of the lung (a neuroendocrine carcinoma). Recent evidence has suggested that expression of pS2 is closely associated with neuroendocrine differentiation in prostate cancer [15]. Colombel et al from in an RT-PCR study found a high expression of pS2 in prostate cancer; however, they found no correlation between with tumour stage or Gleason grade. Our present work [15] indicates that NE differentiation not only correlates with other prognostic markers like grade of the cancer but also has independent prognostic value. Bonkhoff et al [15] noted that pS2 expression was consistently confined to NE differentiation in untreated tumors and in carcinomas that relapsed after hormonal therapy. Our results have similarly shown that 6 out of 9 cancers that have expressed pS2 were also positive for CgA.

## Conclusions

Our results demonstrate that although the expression of pS2 protein was noted in only 1/10th of prostate cancers, it significantly correlates with the histological grade and NE differentiation; both have independent and interdependent prognostic value. There is dearth of data exploring the correlation of pS2 expression and aggressiveness of prostate cancer cell behavior. Limited literature available at present show significant association of pS2 expression with prognosis in prostate cancer, however more work is required to explore the utility of this marker in defining the biological potential of prostate cancer.

## Competing interest

The author(s) declare that they have no competing interests.

## Authors' contributions

MHA, conceived of the idea, wrote the manuscript and conducted clinical part of the study. FA, helped in designing the study and reviewed the draft of the manuscript

NF, helped in conducting study, helped in data collection and analysis. MI, conducted the pathological part of the study. SP, conducted and supervised the pathological aspects of the study and reviewed the manuscript and wrote methods and results related to the pathology. All authors' have read and approve of the final manuscript.

## Pre-publication history

The pre-publication history for this paper can be accessed here:



## Figures and Tables

**Figure 3:**
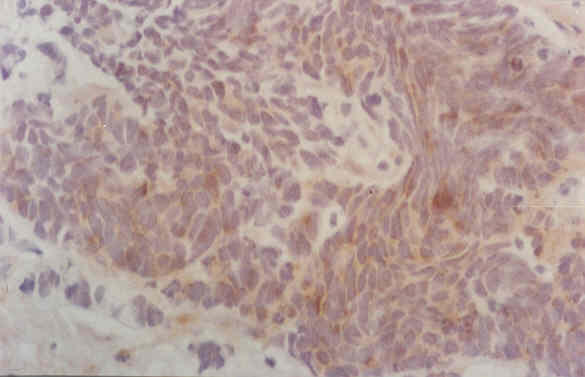
Photomicrograph of prostatic adenocarcinoma stained with polyclonal antibody against CgA. Note prominent cytoplasmic staining within tumour cells. Magnification: 4×

**Figure 5:**
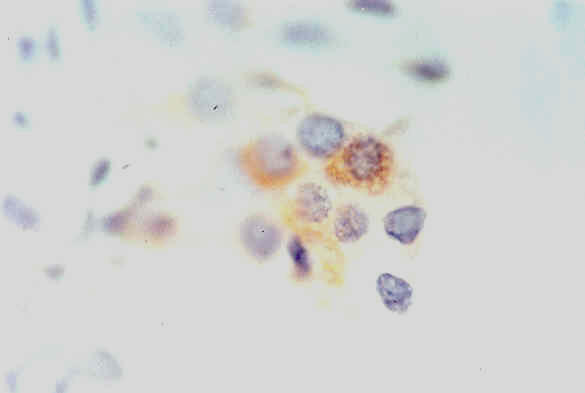
Photomicrograph of prostatic adenocarcinoma stailed with a polyclonal against pS2. Note prominent perinuclear brown staining in tumour cells. Higher magnification (100×).

**Table 1:** Correlation of pS2 expression with CgA expression and Gleason sum (GS)

	**n (%)**	**CgA +ve**	**CgA -ve**	**Mean GS**
pS2 +ve.	9(10)	67%	33%	7.5
pS2 -ve.	86(90)	28%	72%	6.2

+ve. Expression positive

-ve. Expression negative

CgA Chromogranin A

GS Gleeson sum
